# Application of environmental-safe fermentation with *Saccharomyces cerevisiae* for increasing the cinnamon biological activities

**DOI:** 10.1186/s40643-023-00632-9

**Published:** 2023-02-05

**Authors:** Osama M. Darwesh, Aya S. Eweys, Yan-Sheng Zhao, Ibrahim A. Matter

**Affiliations:** 1 Agricultural Microbiology Department, National Research Centre, Cairo, 12622 Egypt; 2 Food Science Department, Faculty of Agriculture, Cairo University, Giza, 12613 Egypt; 3 School of Food and Biological Engineering, Jiangsu University, Zhenjiang, 212013 China

**Keywords:** *S. cerevisiae*, Fermentation, Antioxidant activity, *Cinnamomum cassia*, Antimicrobial, Anti-inflammatory

## Abstract

The effect of fermentation by *Saccharomyces cerevisiae* on biological properties of cinnamon (*Cinnamomum cassia*) was investigated. The study demonstrated that the extract of *S. cerevisiae*-fermented cinnamon (S.C.FC) has antioxidants higher than non-fermented one. The optimum results for antioxidant yield were noted with 10^7^ CFU *S. cerevisiae*/10 g cinnamon and 70 mL of dH_2_O at pH 6 and incubated for 3 d at 35 °C. Under optimum conditions, ABTS, DPPH, and H_2_O_2_ radical-scavenging activity increased by 43.8, 61.5, and 71.9%, respectively. Additionally, the total phenols and flavonoids in S.C.FC were increased by 81.3 and 415% compared by non-fermented one. The fermented cinnamon had antimicrobial activity against *L. monocytogenes, S. aureus*, *E. coli, S. typhi,* and *C. albicans.* Also, the anti-inflammatory properties were increased from 89 to 92% after fermentation. The lyophilized extract of S.C.FC showed positive effect against Huh7 cancer cells which decreased by 31% at the concentration of 700 µg/mL. According to HPLC analysis, *p*-hydroxybenzoic acid, gentisic acid, catechin, chlorogenic acid, caffeic acid, and syringic acid were increased by 116, 33.2, 59.6, 50.6, 1.6, and 16.9%, respectively. Our findings suggest the applicability of cinnamon fermentation using *S. cerevisiae* as a useful tool for processing functional foods to increase their antioxidant and anti-inflammatory content.

**Graphical Abstract:**

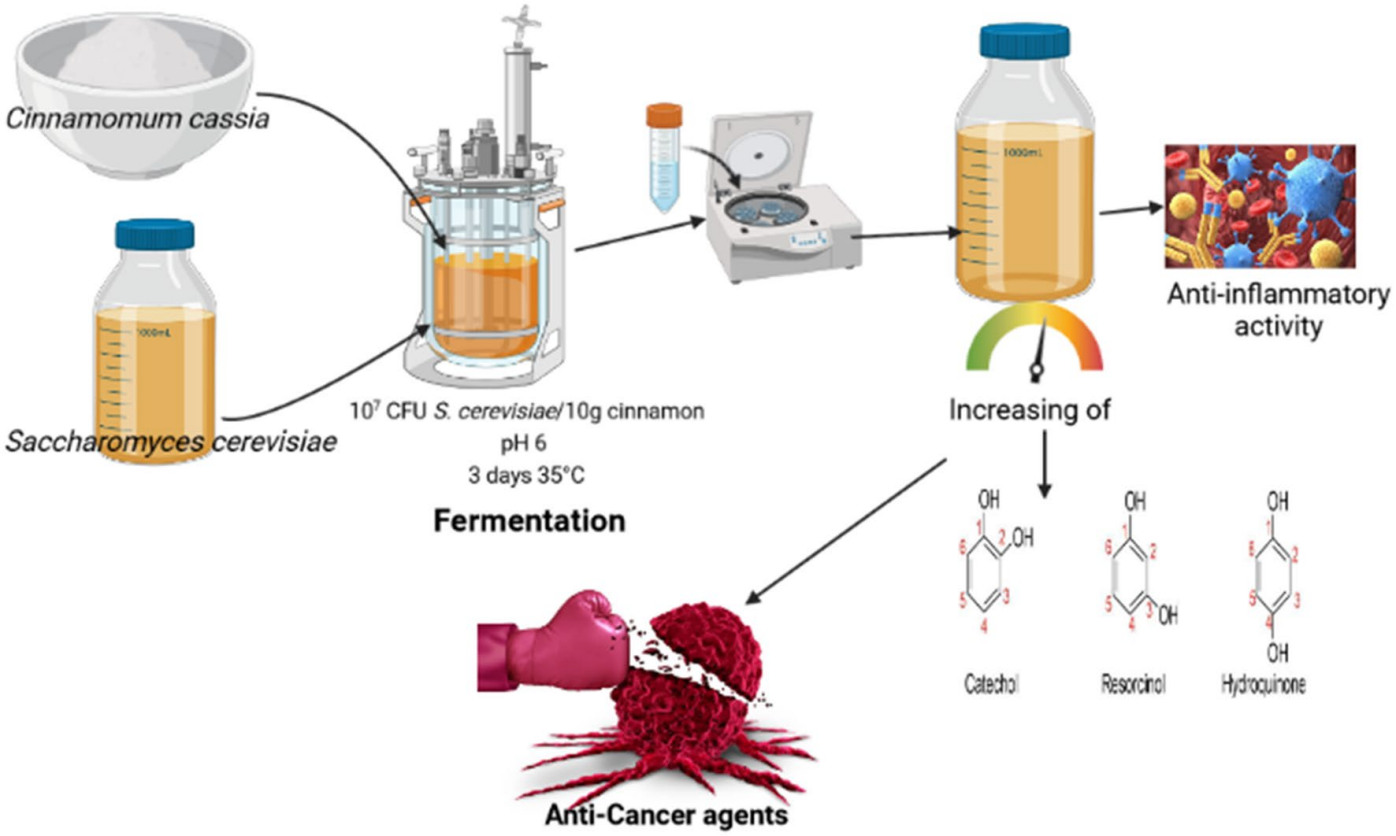

**Supplementary Information:**

The online version contains supplementary material available at 10.1186/s40643-023-00632-9.

## Introduction

Cinnamon as a multi-purpose medicinal plant is widely used around the world as a food additive, hot drink, and condiment. It is produced from the inner bark of tropical tree species belonging to the genus *Cinnamomum* (*Lauraceae* family). The most well-known species in the genus *Cinnamomum* are *C. cassia*, along with *C. verum*, *C. zeylanicum*, and *C. burmannii* (Gutiérrez et al. 2021; Liu et al. 2021; Nunes et al. 2022). Numerous illnesses, including gastrointestinal pain, cancer, infections, and the common cold, can be successfully treated with cinnamon as a supplementary therapy (Hamidpour et al. 2015). According to many reports, cinnamon considered as an antioxidant, analgesic, anti-ulcer, anti-bacterial, anti-allergic, anti-inflammatory, antipyretic, anti-cancer, and anti-diabetic agent (Abdel-Tawwab et al. 2018; Sadeghi et al. 2019**)**.

The antioxidant properties of cinnamon extract are comparable to those of the synthetic antioxidant benchmark butylated hydroxytoluene (BHT). Moreover, it showed protection activity against irradiation-induced lipid peroxidation in liposomes, and managed to quench hydrogen peroxide and hydroxyl radicals (Su et al. 2007; Eweys et al. 2022). Also, it has been reported that cinnamon improves the insulin sensitivity index by enhancing insulin resistance, and thus can reduce diabetic syndrome by normalizing pancreatic function (Li et al. 2013). Cinnamon volatile oils are highly effective as antimicrobials against many multidrug-resistant bacteria and fungi. Its anti-bacterial activity has been reported to be more effective than some common antibiotics (e.g., ampicillin, chloramphenicol, and streptomycin) (El Atki et al. 2019). The nature of food processing has a positive or negative impact on its contents of biologically active compounds (including antioxidants). Some treatments increase the extractability of some compounds or cause structural transformations in others. For example, Ravichandran et al. (2013) reported that microwave, roasting, and boiling provided better extractability. The antioxidant activity of red beets (up to threefold) was significantly increased due to the polyphenol content.

Fermentation converts the conjugated phenolic compounds to more hydroxyl groups which cause changes in antioxidant activity during fermentation (Wang et al. 2018; Eweys et al. 2022). Zhao et al. (2021a) demonstrated how the structural collapse of cereal cell walls, which allowed for the creation and liberation of a variety of bioactive chemicals, allowed microbes to change plant elements. Also, Hur et al. (2014) confirmed that enzymes, such as cellulases, inulinase, tannase, amylase, and glucosidase, could be produced during fermentation, and can breakdown the plant cell wall producing bioactive compounds.

The yeast,* Saccharomyces cerevisiae*, is a useful tool for most aspects of basic research. Despite efforts to find new microbes, *S. cerevisiae* continues to be one of the most fermentation bioagent (Klopp et al. 2021). The biotechnological use of *S. cerevisiae* is based on its unique biological properties, namely its fermentation capacity (Parapouli et al. 2020). In particular, three characteristics are very important for some industrial applications of *S. cerevisiae*, its capability of withstanding stressful conditions, rapid growth, and fermentation efficiency (Andrietta et al. 2007). Also, there are two important properties of *S. cerevisiae* that are extremely essential for industrial applications: its tolerance to high sugar concentrations, and the production of fragrant and aromatic chemicals (Parapouli et al. 2020). The improvement of *S. cerevisiae* to the organoleptic characteristics can be returned to its ability to produce flavor compounds, such as aldehydes, esters, and alcohols (Menezes et al. 2016; Magalhaes et al. 2017). In biological systems, phenolic substances exhibited peroxide decomposition and free radical inhibition (Aryal et al. 2019). Furthermore, numerous studies have shown that flavonoid consumption improves heart performance (Banjarnahor 2014). As a result, recent research emphasizes the functional activity of flavonoids, and phenolics as antioxidants against oxidative stress (Banjarnahor 2014; Aryal et al. 2019). For that, the present work is a scientific contribution to study the effect of *S. cerevisiae* fermentation on the biological properties of cinnamon. Hopefully, the results of this study are valuable in this field.

## Materials and methods

### Materials, reagents, and media

Cinnamon (*C. cassia*) powder was purchased from a local store in Egypt (Kirkland organic ground Saigon cinnamon, Vietnam). Lactobacilli MRS Broth (NEOGEN, NCM0079B), Nutrient broth, and Nutrient agar media were obtained from HiMedia Leading BioSciences Company, India. All chemicals and reagents used were of analytical grade and purchased by lab suppliers from Sigma-Aldrich, VWR chemicals and Merk, India.

### Saccharomyces cerevisiae strain

*Saccharomyces cerevisiae* was isolated and purified from the commercial compressed baker's fresh yeast available in the local markets in Egypt. Briefly, 1 g of compressed baker's fresh yeast were added to 90 mL of sterilized saline water, and various serial dilutions were made (1 mL of previous culture/9 mL water to make new dilution). One mL from different dilutions was inoculated on the surface of nutrient agar plates and then incubated at 35 ± 2 ℃ for 24–48 h. A separated single colony was picked up and re-streaked on new nutrient agar plates for purification. The purified isolate was subjected to microscopic and cultural examination according to the protocol of Kurtzman et al. (2011) and Khattab et al. (2016) to confirm that it is *S. cerevisiae*. The cultural examinations include the growth pattern on solid and liquid media as well as the ability to use glucose, galactose, maltose, lactose, sucrose, and ethanol individually as the sole carbon source. The purified yeast was maintained on nutrient agar slants at refrigerator (4 °C) for further experiments.

### Fermentation technology of cinnamon aqueous solution

Fermentation starter was prepared by cultivation of *S. cerevisiae* strain loop in 250-mL conical flasks containing 50 mL of Nutrient broth and incubated for 24 h at 35 ℃ on a rotary shaker at 120 rpm. After incubation, cells were harvested by centrifugation (5600 g for 10 min) and then re-suspended in a sufficient volume of sterile distilled water to obtain a cell density of 10^7^ CFU/mL. The immediately obtained *S. cerevisiae* suspension was used as a fermentation initiator with a specified volume according to the scheme. Fermented cinnamon was prepared according to the method of Eweys et al. (2022) with some minor modifications. In brief, 10 g of sterilized cinnamon powder was mixed with 70 mL of sterilized distilled water, and then, specific volume of *S. cerevisiae* as inoculum was added. The fermentation mixture was incubated at 35 ℃ on a rotary shaker at 120 rpm. Some important fermentation factors were included to study their effect on antioxidant production, namely inoculum concentration, incubation period, and initial pH. The cell concentration of the *S. cerevisiae* inoculum used for fermentation was 10^0^, 10^2^, 10^3^, 10^5^, 10^7^, and 10^9^ CFU/ 10 g of cinnamon. While the incubation periods were 1, 2, 3, 4, and 5 days, and the initial pH was adjusted at values of 4, 5, 6, 7, and 8 using 1 N of HCl or NaOH. The unfermented (cinnamon sample without *S. cerevisiae* inoculation) was used as a control in this experiment. The counts of *S. cerevisiae* in the fermented samples were enumerated by pour plate technique using nutrient agar medium.

### Extraction of fermented cinnamon

The aqueous phase obtained after the centrifugal removal (5600 g for 10 min) of cells and sediment of the cinnamon fermentation mixture was used as the “extract”. For comparison, non-fermented cinnamon was subjected to a similar extraction process using only distilled water. The aqueous extracts of fermented and non-fermented cinnamon were freeze-dried at – 50 ℃ for 48 h (Labconco freeze dryer, Console, USA). Freeze-dried cinnamon extracts were analyzed for their total phenol and flavonoid content, antimicrobial activity, anti-inflammatory activity, and anti-cancer activity. HPLC and TLC analyses also have been conducted for the lyophilized extracts.

### Determination of antioxidant activity

The antioxidant samples were prepared by dissolving 0.2 mg of the lyophilized cinnamon extract in 1 mL of distilled water, in which DPPH, ABTS, and hydroxyl radical-scavenging activity (%) were measured.

### DPPH radical-scavenging assay

The antioxidant activity of cinnamon was measured using 2, 2-diphenyl-1-picrylhydrazyl (DPPH˙) method described by Darwesh et al. (2022) with some modifications. Briefly, a portion of 0.1 mL sample was added to 3.9 mL of freshly prepared DPPH solution (22 mg of DPPH in 50 mL methanol). The mixture was vortexed for 30 s then kept in a dark place at room temperature for 30 min. The decolorization of DPPH reaction mixture was measured at 515 nm using a UV–Vis spectrophotometer (JASCO serial No. A114761798, Japan). The DPPH free radical-scavenging activity (%) was calculated as the percentage of absorbance decrease.

### ABTS radical-scavenging assay

According to Mohdaly et al. (2010) with some modifications, an equal volume of 2.4 mmol/L potassium persulfate was mixed with 7 mM ABTS and then incubated at room temperature for 12–16 h in the dark. The ABTS^+^ solution was diluted with distilled water to obtain initial absorbance of 0.70 ± 0.02 at 734 nm. A 0.25 mL of 60-fold diluted sample was added to 0.75 mL of distilled water and 1 mL of 2,2′-azino-bis(3-ethylbenzothiazoline-6-sulfonate diammonium) (ABTS^+^) solution, and then incubated at room temperature for 7 min. The absorbance decrease was recorded at 734 nm and ABTS^+^ scavenging effect (%) was calculated as the percentage of absorbance decrease.

### Hydroxyl radical-scavenging activity assay

Hydroxyl radical-scavenging activity was determined by the method presented by Bhattaram et al. (2002) with some modifications. A volume of 0.3 mL sample was added to 0.9 mL of 50 mM phosphate buffer (pH = 7.4) and 1.8 mL of H_2_O_2_ solution (2 mM). The mixture was vortexed and kept at room temperature for 10 min. The absorbance decrease was measured at 230 nm and the scavenging activity (%) was calculated as the percentage of absorbance decrease.

### Determination of total phenolic contents

The total phenolic content (TPC) was determined according to Khan et al. (2018) and Eweys et al. (2022) with minor modifications. A 0.5 mL of cinnamon extract solution (2 mg lyophilized extract/mL DW) was mixed with 0.5 mL of 10% Folin–Ciocalteu’s reagent diluted in 13 mL distilled water. After that, 2.5 mL of 7% Na_2_CO_3_ solution was added followed by mixing. The reaction mixture was incubated at room temperature for 2 h in the dark. The absorbance was measured at 760 nm. Total phenolic content was calculated by extrapolating a calibration line constructed with gallic acid as standard solution (Additional file 1: Fig. S1). The total phenolic content was expressed as mg gallic acid equivalent per gram dry weight extract (mg GAE/ g lyophilized extract).

### Determination of total flavonoid contents

The total flavonoids content was determined according to method described by Khan et al. (2018) with some modifications. In brief, 0.5 mL of cinnamon extract solution (2 mg lyophilized extract/mL DW) was mixed with 2.5 mL of distilled water, 1 mL of potassium acetate (1 M), and 1 mL of 10% aluminum chloride. The total flavonoids’ content was estimated by extrapolating a calibration line constructed with quercetin solution (Additional file 1: Fig. S2). The absorbance of the reaction was measured at 415 nm using UV–Vis spectrophotometer. The total flavonoid content was stated in terms of quercetin equivalent (mg QE/ g of lyophilized extract).

### Evaluation of antimicrobial activity

The antimicrobial activity of the lyophilized aqueous cinnamon extract (0.2 g/ mL DW) was determined using the agar well diffusion method reported by Sultan et al. (2016) with some modifications. The tested microorganisms were Gram-negative bacteria (*E. coli* ATCC-25922 and *Salmonella typhi* ATCC-15566), Gram-positive bacteria (*Listeria monocytogenes* ATCC-35152 and *Staphylococcus aureus* ATCC-47077), and yeast (*Candida albicans* ATCC-10231). All test strains were obtained from the American Culture Collection (ATCC, Rockville, MD, USA). The tested strains were cultured in nutrient broth medium for 24 h; after that, an adequate volume (100 µL from each) was spread onto the surface of nutrient agar plates. The wells (7 mm diameter) were mined on the inoculated plates and 75 µL of the previous DW–cinnamon mixture was added to the well (Darwesh et al. 2019). The plates were incubated at 35 ± 2 ℃ for 16–24 h, and then, the clear zones have been measured. The antimicrobial activities calculated according to the obtained inhibition zones on agar plates.

### Determination the effect of fermentation technology on the anti-inflammatory activity of cinnamon

The human red blood cell (HRBC) membrane stabilization method was applied to test the in vitro anti-inflammatory activity of cinnamon sample (Vana and Botting 1995). Blood was collected from one healthy volunteer. The collected blood was mixed with equal volume of sterilized Alsever′s Solution (2% dextrose, 0.8% sodium citrate, 0.05% citric acid, and 0.42% sodium chloride in water). The blood was centrifuged at 2016 g for 20 min and packed cells were separated. The packed cells were washed with isosaline (0.85%, pH 7.2) and a 10% *v*/*v* suspension was made with isosaline. The HRBC suspension was used for the estimation of anti-inflammatory activity. One milliliter of sample and Diclofenac sodium (as positive control) were separately mixed with 1 mL of phosphate buffer (0.15 M, pH 7.4), 2 mL of hyposaline (0.36%) and 0.5 mL of HRBC suspension. Instead of sample, 2 mL distilled water was used as the negative control. All the assay mixture was incubated at 37 °C for 30 min and centrifuged at 2016 g for 20 min. The supernatant liquid was decanted and the hemoglobin content in the supernatant solution was estimated using spectrophotometer at 560 nm. Percentage of hemolysis was estimated by assuming the hemolysis produced in the control as 100%. The percentage hemolysis was calculated using the following formula:

$${\text{\% Hemolysis}} = \left( {\frac{OD\, sample}{{OD\, control}}} \right)*100.$$

The percentage of HRBC membrane stabilization or protection was calculated by the following formula:

$${\text{\% Protection}} = 100 - \left( {\frac{OD\, sample}{{OD\, control}}} \right)*100.$$

### Anticancer activity evaluation and cytotoxicity determination of cinnamon

Huh7 (liver cancer cell line) and Wi38 cells (human lung fibroblast cell line) were purchased from VACSERA (the Holding Company for Biological Products and Vaccines) at Giza governorate, Egypt and the procedures were done in Cairo University Research Park (CURP). The test was done according to neutral red uptake assay (Repetto et al. 2018; Hussein et al. 2019). Trypsinized cells were sub-cultured into 25cm^2^ tissue culture flasks. A 5 × 10^5^ cells were cultured in each flask containing 7 mL of complete Dulbecco’s modified eagle medium (DMEM) supplemented with 1% antibiotic solution (100 U/mL penicillin and 100 μg/mL streptomycin) and 10% of fetal bovine serum and incubated at 37 ± 1 °C. All culture reagents were purchased from Lonza supplier in Egypt. Huh7 cells as cancer cells were divided as follows: Group 1 served as untreated control without any treatment, Group 2 cells inoculated with freeze-dried fermented cinnamon extract at 100, 300, 500, and 700 µg/mL, and Group 3 cells inoculated with freeze-dried non-fermented cinnamon extract at 100, 300, 500, and 700 µg/mL. Also, Wi38 cells as normal cells were divided as mentioned with Huh7 cells. The cells were exposed to neutral red dye (4 mg/mL) in serum free DMEM after treatment periods for 3 h. The cells were washed with phosphate-buffered saline (PBS) and then de-stained using de-staining solution (50% EtOH and 5% glacial acetic acid in distilled water). The absorbance of residues neutral red dye was measured at 540 nm using spectrophotometer. Each treatment was done in triplicates after 24 h of incubation.

### Thin-layer chromatography for characterization of cinnamon extracts

Active components of lyophilized fermented and non-fermented cinnamon extracts were characterized by thin-layer chromatography (TLC). TLC silica gel 60 F_254_ plates (Merck KGaA, Darmstadt, Germany) were prepared in system of ethyl acetate: hexane at a ratio of 1:1 *v*/*v* using TLC paper covered with silica gel as stationary phase (Utchariyakiat et al. 2016). They were emerged in the working solvent system as mobile phase. The TLC plate was visualized under UV light (254 and 366 nm).

### HPLC analysis for characterization of cinnamon extracts

High-performance liquid chromatography (HPLC) analysis was carried out according to Kim et al. (2006) using Agilent Technologies 1100 series liquid chromatograph equipped with an autosampler and a diode-array detector. The analytical column was an Eclipse XDB-C18 (150 × 4.6 µm; 5 µm) with a C18 guard column (Phenomenex, Torrance, CA). The mobile phase consisted of acetonitrile (solvent A) and 2% acetic acid in water (*v*/*v*) (solvent B). The flow rate was kept at 1 mL/min for a total run time of 60 min and the gradient program was as follows: 100 to 85% B in 30 min, 85 to 50% B in 20 min, 50 to 0% B in 5 min, and 0 to 100% B in 5 min. The injection volume was 20 µL and peaks were monitored simultaneously at 280 and 320 nm for the benzoic acid and cinnamic acid derivatives, respectively, as well as 360 nm for flavonoids. All samples were filtered through a 0.45 µm acrodisc syringe filter (Gelman Laboratory, MI) before injection. Peaks were identified by congruent retention times and UV spectra and compared with those of the standards.

### Statistical analysis

All analyses were performed in triplicate and data reported as the mean ± standard deviation (SD). Results were processed by One-Way ANOVA: analysis of variance using the statistical software of Statistix 8.1 (Analytical Software, Tallahassee, FL, USA) using LSD’s test for pairwise comparison.

## Results and discussion

### S. cerevisiae strain, isolation, purification, and identification

Choosing the right microbial strain contributes significantly to the success of fermentation process, types, and quantities of emerging products. In this context, *S. cerevisiae* is one of the most industrially known yeast strains for large-scale fermentation in a long time (Walker and Stewart 2016). As that, *S. cerevisiae* was selected as the bioagent for cinnamon powder fermentation and the potential to influence its yield of antioxidants and other vital compounds*.* The isolate, *S. cerevisiae*, was obtained by purification of the commercial compressed baker’s fresh yeast available in the local markets in Egypt. The purified yeast underwent characterization and morphological identification to confirm its identity as *S. cerevisiae*.

**Fig. 1:**
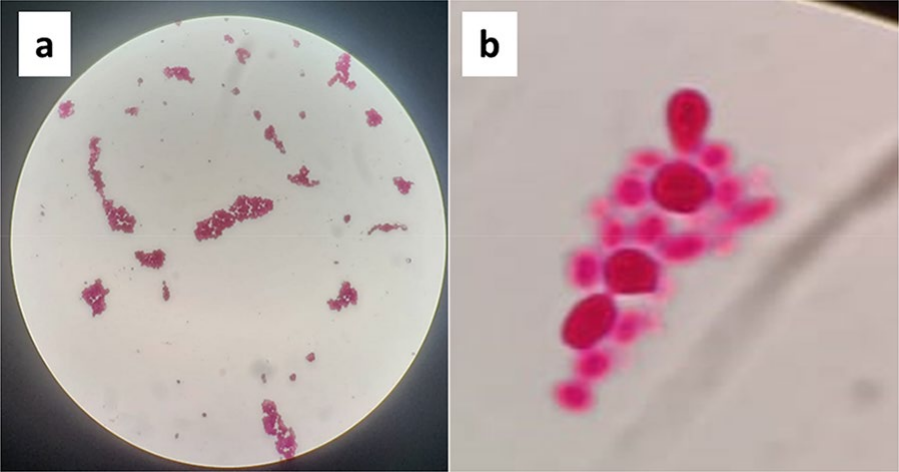
Appearance of a *S. cerevisiae* isolate under a light microscope using 20X (**a**) and 100X (**b**) magnification lenses

The developed single colony on the surface of nutrient agar plate (after being incubated at 35 ± 2 ℃ for 24–48 h) appeared as smooth and raised with a creamy color. The color of the precipitated cells after similar incubation period in the nutrient broth medium was white. Microscopically, the cells were ovoidal and globose with multilateral budding (Fig. 1). On the other hand, the isolate showed well assimilation to glucose, maltose, and sucrose but not lactose, and glycerol. The results of the biochemical tests and the morphological characteristics of the isolated yeast confirm that it is *S. cerevisiae* according to Khattab et al. (2016).

### Influence of S. cerevisiae fermentation on the antioxidant activity of cinnamon

The present study aimed mainly to improve the antioxidant activity of cinnamon by fermentation with the edible probiotic yeast, *S. cerevisiae*. Fermentation products are expected to be affected by the number of microorganisms involved in the fermentation, the fermentation time, and initial pH of the fermentation mixture. Hence, for such investigations, *S. cerevisiae* was applied to cinnamon solution to determine their ability to enhance antioxidant substances production.

The optimum yeast concentration must be used to provide the optimum fermentation properties. Fermentation is an enzymatic process; hence, the low concentration of yeast will cause slow fermentation (Gunawan et al. 2018). Using a high yeast concentration can cause yeast competition and fighting each other, leading to growth inhibition (Hibbing et al. 2010). Therefore, it is very important to use the optimum yeast concentration as an inoculum for the fermentation process. In this context, the antioxidant productivity of cinnamon based on fermentation with different concentrations of *S. cerevisiae* was investigated. Five cell concentrations of yeast were applied for cinnamon fermentation; 10^2^, 10^3^, 10^5^, 10^7^, and 10^9^ CFU/10 g of cinnamon. A 10^0^ CFU/ 10 g of cinnamon was used as a control.

The antioxidant activity was recorded (and calculated) as a percentage of ABTS radical-scavenging activity, DPPH radical-scavenging activity, and H_2_O_2_ scavenging activity. The results of antioxidant activities after aqueous extraction were measured for fermented and non-fermented cinnamon and are illustrated in Table (1). The obtained results revealed that the concentration of *S. cerevisiae* cells in fermentation mixture for cinnamon influences its antioxidant yield. Regarding DPPH scavenging activity, the results of antioxidant yield due to initial inoculum yeast concentration of 10^9^ CFU/ 10 g of cinnamon were significantly the highest compared to lower other initial inoculant concentrations and the control. However, the inoculum concentration of 10^7^ CFU/ 10 g of cinnamon showed significantly higher antioxidant activity than all other lower concentrations, but without significance compared to the control group. The antioxidant activity expressed with ABTS was highest when the initial inoculum was 10^7^ CFU/10 g of cinnamon; although it was not significant with those of 10^5^ and 10^9^ CFU/10 g of cinnamon. In addition, there were no significant differences between the ABTS results for non-fermented cinnamon and the fermented with initial yeast concentrations of 10^2^ and 10^5^ CFU/10 g cinnamon. On the contrary, the antioxidant activity of cinnamon expressed as H_2_O_2_ scavenging activity scored the lower value when the applied yeast inoculant was 10^9^ CFU/10 g cinnamon. The results showed that the concentration of yeast inoculum at 10^7^ CFU/10 g cinnamon was the most suitable in terms of high antioxidant production during the fermentation of cinnamon by *S. cerevisiae*. The low cell concentration gave lower value as compare to without fermentation, it may due to the microbial culture is not enough to ferment the cinnamon (decomposition of polyphenols or complex compounds to phenols or simple one) and the microbial cells uses the simple compounds like phenols for growth, followed decreasing in antioxidant activity.

**Table 1:** Effect of different *S. cerevisiae* inoculum concentrations (as CFU/ 10 g cinnamon) used for cinnamon fermentation on its antioxidant activity at pH 7, 35 ˚C for 3 d

CFU/ 10 g cinnamon	ABTS radical-scavenging activity %	DPPH radical-scavenging activity %	Hydroxyl radical-scavenging activity %
0.0	29.49^c^	66.93^b^	61.62^ab^
10^2^	31.41^bc^	47.81^e^	33.33^bc^
10^3^	33.87^bc^	51.17^d^	40.81^bc^
10^5^	36.43^ab^	53.69^c^	55.96^ab^
10^7^	40.80^a^	68.87^b^	69.90^a^
10^9^	40.69^a^	73.60^a^	25.05^c^

Where the data are the means of triplicate experiments; ^a, b, c, d^ values in the same column with different superscript letters are significantly different (P < 0.05). ^a^ value is the highest antioxidant activity and ^e^ value is the lowest antioxidant activity in the same column

For the fermentation process, the length of time that microorganisms are in contact with the substrate is crucial. Therefore, it is crucial to provide the fermentation process enough time to produce the targeted components (Gunawan et al. 2018; Aung 2022). Hence, the effect of different yeast fermentation periods on the production of antioxidants from cinnamon was studied to determine the sufficient time to obtain the highest yield. Five different incubation periods, 1, 2, 3, 4, and 5 days, were used for cinnamon fermentation. Following various incubation times, the antioxidant activities of aqueous extract samples of *S. cerevisiae*-fermented cinnamon were examined and the results of scavenging activities expressed by ABTS, DPPH, and H_2_O_2_ radicals are shown in Table (2). In general, the antioxidant yield of fermented cinnamon increased until the third incubation day and then decreased on the fourth and fifth days. The increase in fermentation-induced antioxidants during the first 3 days can be attributed to the activity of *S. cerevisiae* in converting the complex compounds in cinnamon into simple, easy-to-extract forms. Whereas the decrease in antioxidant activity after a relatively long incubation period can be attributed to microbial competition, or microbial decomposition of some bioactive compounds (Hibbing et al. 2010). Based on the high antioxidant yield of cinnamon by fermentation during 3 days with yeast, this period was used as an incubation period for the following experiments.

**Table 2:** The antioxidant activity of cinnamon as scavenging activity (%) during different fermentation periods by *S. cerevisiae* at 10^7^ CFU, pH 7, 35 ˚C

Fermentation time (Day)	ABTS radical-scavenging activity %	DPPH radical-scavenging activity %	Hydroxyl radical-scavenging activity %
1	20.58^c^	56.23^c^	18.03^b^
2	38.74^ab^	74.45^ab^	48.85^ab^
3	46.76^a^	79.13^a^	84.70^a^
4	38.55^ab^	69.11^b^	50.73^ab^
5	34.69^b^	43.85^d^	31.66^b^

Where the data are the means of triplicate experiments; samples in the same column with different superscript letters are significantly different (P < 0.05)

^a^ value is the highest antioxidant activity, ^b ^value is the medium antioxidant activity and ^c^ value is the lowest antioxidant activity in the same column

The microbe's enzymatic system is directly impacted by the pH of the fermentation medium, which has an impact on how will the microbe can grow and create different byproducts (Namasivayam et al. 2011; Zhao et al. 2021b). Furthermore, changing the pH below or above the optimum value may result in the formation of different (sometimes undesirable) fermentation products. In addition, it may affect the bio-transformation processes of materials either negatively or positively (Lee et al. 2021). The effect of the difference in initial pH of *S. cerevisiae*–cinnamon mixture on the antioxidant yield after 3 days of fermentation was studied. The initial pH values under study ranged from 4 to 8, and the values of the antioxidants produced in the aqueous extract of fermented cinnamon are represented in Table 3. The cinnamon fermented by *S. cerevisiae* had the maximum antioxidant activity measured by ABTS, DPPH, and H_2_O_2_ when the initial pH was 7. Although cinnamon initially fermented at pH 7 had the highest levels of antioxidants, its antioxidant activity was not significantly different from that fermented at pH 6. It is noted that the original pH of the cinnamon–yeast mixture was close to 6 (at 10 g of cinnamon and 70 mL of distilled water). Therefore, the economic cost of adjusting the pH to 7 at the industrial or semi-industrial level should be taken into account, since there are no significant differences between it and the results of the pH 6 regarding the yield of antioxidants from fermented cinnamon.

**Table 3:** The antioxidant activity of cinnamon–*S. cerevisiae* fermentation mixture at different initial pH values with 10^7^ CFU, 35 ˚C for 3 d

pH value	ABTS radical-scavenging activity %	DPPH radical-scavenging activity %	Hydroxyl radical-scavenging activity %
4	47.05^b^	83.18^c^	26.67^c^
5	51.79^ab^	84.38^bc^	44.24^bc^
6	55.65^ab^	89.86^ab^	47.27^abc^
7	61.06^a^	94.87^a^	65.86^a^
8	43.48^b^	89.09^abc^	55.96^ab^

Where the data are the means of triplicate experiments; samples in the same column with different superscript letters are significantly different (P < 0.05). ^a^ value is the highest antioxidant activity, ^b^ value is the medium antioxidant activity and ^c^ value is the lowest antioxidant activity in the same column

### Optimized cinnamon fermentation conditions with S. cerevisiae

The current study focuses on the effect of fermentation by *S. cerevisiae* on increasing the antioxidant productivity from cinnamon. According to earlier findings, adjusting the fermentation conditions can increase the amount of antioxidants produced by cinnamon. Optimal conditions include a 3-day incubation period at 35 °C, an initial pH of 6, and an initial yeast density of 10^7^ CFU/10 g cinnamon. Aqueous extracts of fermented cinnamon (under ideal conditions) and non-fermented cinnamon that had been freeze-dried were evaluated. The characteristic covers antioxidant activities, total phenols and flavonoids, antimicrobial activity, anti-inflammatory, anti-cancer and cytotoxic activities, TLC, and HPLC were analyzed.

### Antioxidant activities, total phenols, and flavonoids

Indicators of DPPH, ABTS, and H_2_O_2_ were used to assess the antioxidant activity of lyophilized cinnamon extracts. The results showed that the lyophilized extract of fermented cinnamon scavenged ABTS, DPPH, and hydroxyl radicals considerably better than non-fermented cinnamon (Table 4). According to the ABTS radical-scavenging activity, cinnamon fermented with *S. cerevisiae* was 43.8% better than non-fermented cinnamon. Based on the DPPH test for radical-scavenging activity, fermented cinnamon had 61.5% more antioxidants than unfermented. While the percentage of increase when taking into account the measure of H_2_O_2_ radical-scavenging activity for antioxidants was about 71.9% in fermented cinnamon than in non-fermented cinnamon. On the other hand, fermentation was found to enhance the content of phenols and total flavonoids of cinnamon extract. The obtained result (Table 4) confirms the increase of total extracted phenols and flavonoids in *S. cerevisiae*-fermented cinnamon more than non-fermented one by 81.3 and 415%, respectively. Natural flavonoid and phenolic compounds are plant secondary metabolites which hold an aromatic ring bearing at least one hydroxyl group (Tungmunnithum et al. 2018). It is well clear that fermentation of cinnamon causes an increase in the contents of total flavonoid and phenolic contents in its lyophilized extract. This incensement could be attributed to the bio-transformation of some cinnamon’s components or the breakdown of cell walls, which helps produce a variety of antioxidant chemicals (Hur et al. 2014).

**Table 4:** Antioxidant activity, total phenols, and flavonoids contents of lyophilized fermented and non-fermented cinnamon

Radical scavenging activity	Non-fermented cinnamon	Fermented cinnamon
ABTS radical-scavenging activity (%)	24.20^b^	34.81^a^
DPPH radical-scavenging activity (%)	42.72^b^	68.98^a^
Hydroxyl radical-scavenging activity (%)	21.52^b^	36.78^a^
Total phenolic content (mg GAE/L extract)	8.15^b^	14.78^a^
Total flavonoids content (mg QE/L extract)	0.59^b^	3.04^a^

Where the data are the means of triplicate experiments; samples in the same row with different superscript letters are significantly different (P < 0.05). ^a^ value is the highest scavenging activity, and ^b^ value is the lowest scavenging activity in the same row

### Antimicrobial and anti-inflammatory activity evaluation

**Fig. 2:**
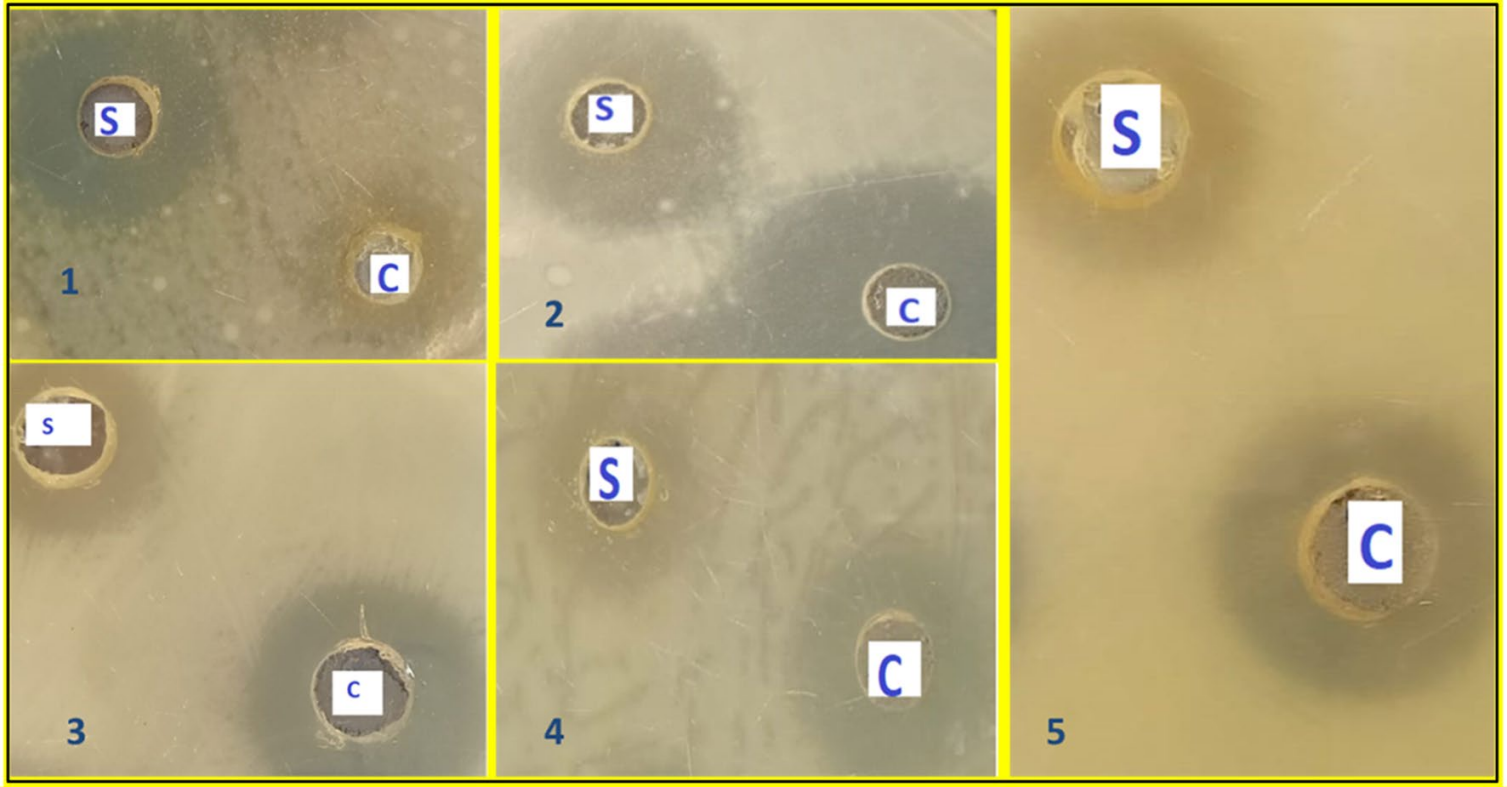
Comparison between the inhibition zones of the lyophilized extracts of fermented and non-fermented cinnamon against different pathogenic microorganisms: (S) fermented sample by *S. cerevisiae*; (C) non-fermented sample; (1) *E. coli*; (2) *S. typhi*; (3) *S. aureus*; (4) *L. monocytogenes* and 50 *C. albicans*

Many herbs and spices have antimicrobial properties that offer them therapeutic value in medicine. They can also be used as food ingredients to extend the shelf life of food and protect it against food poisoning bacteria. The therapeutic use of cinnamon includes its natural anti-bacterial activity against many pathogens such as *Moraxella catarrhalis* that can cause respiratory system infections (Rasheed and Thajuddin 2011; Nabavi et al. 2015). Fermentation is expected to positively or negatively affect cinnamon's antimicrobial activity. It was therefore important to study the antimicrobial efficacy of lyophilized cinnamon extract and the effect of this activity in the case of *S. cerevisiae*-fermented cinnamon. Gram-positive bacteria *L. monocytogenes* and *S. aureus*, Gram-negative bacteria *E. coli* and *S. typhi*, and the yeast *C. albicans* were used in the evaluation. The applied concentration, that is, 200 mg/mL of lyophilized fermented and non-fermented cinnamon extract, showed a positive effect against all studied pathogens. However, fermented cinnamon extract showed lower inhibition than non-fermented cinnamon (measured by diameter of inhibition) against all tested bacteria (Table 5 and Fig. 2). The fermentation cause around 10.5 to 26.6% decrease in the antimicrobial activity of cinnamon extract. The reduced antimicrobial activity of fermented cinnamon extract may be attributed to the hydrolysis of some antimicrobial components of cinnamon, such as cinnamaldehyde and eugenol to their derivatives (Tadasa 1977; Nabavi et al. 2015). It should be noted that cinnamaldehyde degrades to cinnamic acid, which has higher free radical-scavenging properties than cinnamaldehyde besides its anti-tuberculosis and anti-inflammatory properties (Suryanti et al. 2018). In addition, vanillin and ferulic acid are produced during the breakdown of eugenol (Tadasa 1977). This vanillin product has been previously shown to have stronger antioxidant activity than ascorbic acid, trueox in ABTS( +)-scavenging, and oxygen radical absorption capacity (ORAC) assays (Tai et al. 2011).

**Table 5:** Inhibition zone diameters of fermented and non-fermented lyophilized cinnamon extracts against some common pathogens

Microorganism strain	Inhibition zone of fermented cinnamon (mm)	Inhibition zone of non-fermented cinnamon (mm)
*E. coli*	22	26
*S. typhi*	30	38
*L. monocytogenes*	23	26
*S. aureus*	20	23
*C. albicans*	19	21

**Fig. 3:**
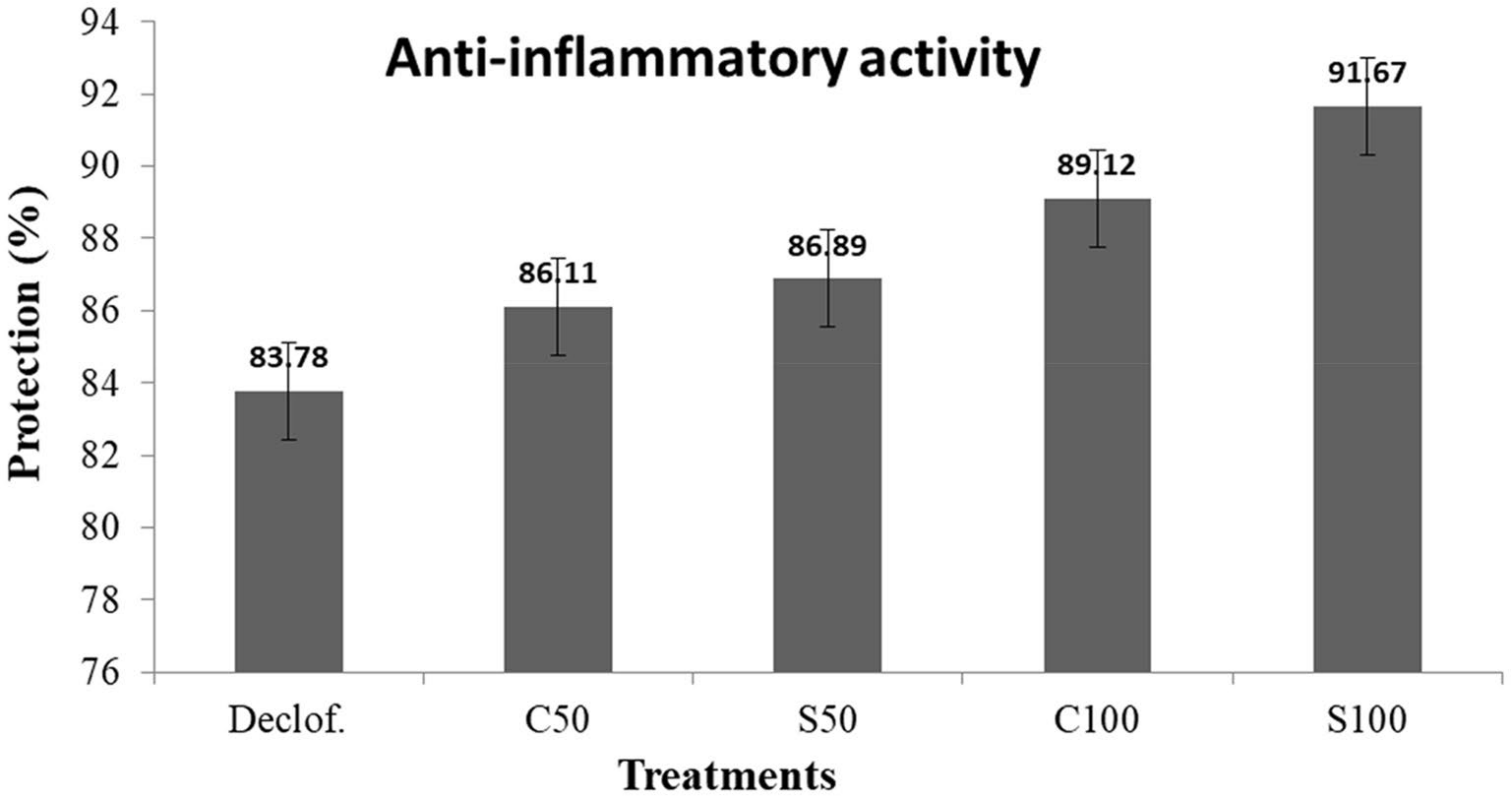
The effect of fermentation by *S. cerevisiae* on anti-inflammatory properties of C. cassia extract: Declof.—non-steroidal anti-inflammatory and phenylacetic acid derivative drug (standard at concentration of 2000 μg/mL); C50—non-fermented cinnamon at 50 ppm; S50—fermented cinnamon at 50 ppm; C100—non-fermented cinnamon at 100 ppm; and S100—fermented cinnamon at 100 ppm

In case of anti-inflammatory properties of *C. cassia* extract after fermentation by *S. cerevisiae*, the cinnamon powder was fermented and evaluated its anti-inflammatory properties comparing with non-fermented one and diclofenac as a non-steroidal anti-inflammatory and phenylacetic acid derivative drug (standard at concentration of 2000 μg/mL). The obtained results (Fig. 3) indicated that increasing the anti-inflammatory properties of cinnamon after fermentation by *S. cerevisiae* from 89 to 92% of human red blood cells’ protection from agglomeration. This may be returned to available of some compounds like cinnamic acid (Godoy et al. 2000; Suryanti et al. 2018), vanillin (Kim et al. 2019), or ferulic acid (Liu et al. 2022).

### Anticancer activity and cytotoxicity evaluation

**Fig. 4:**
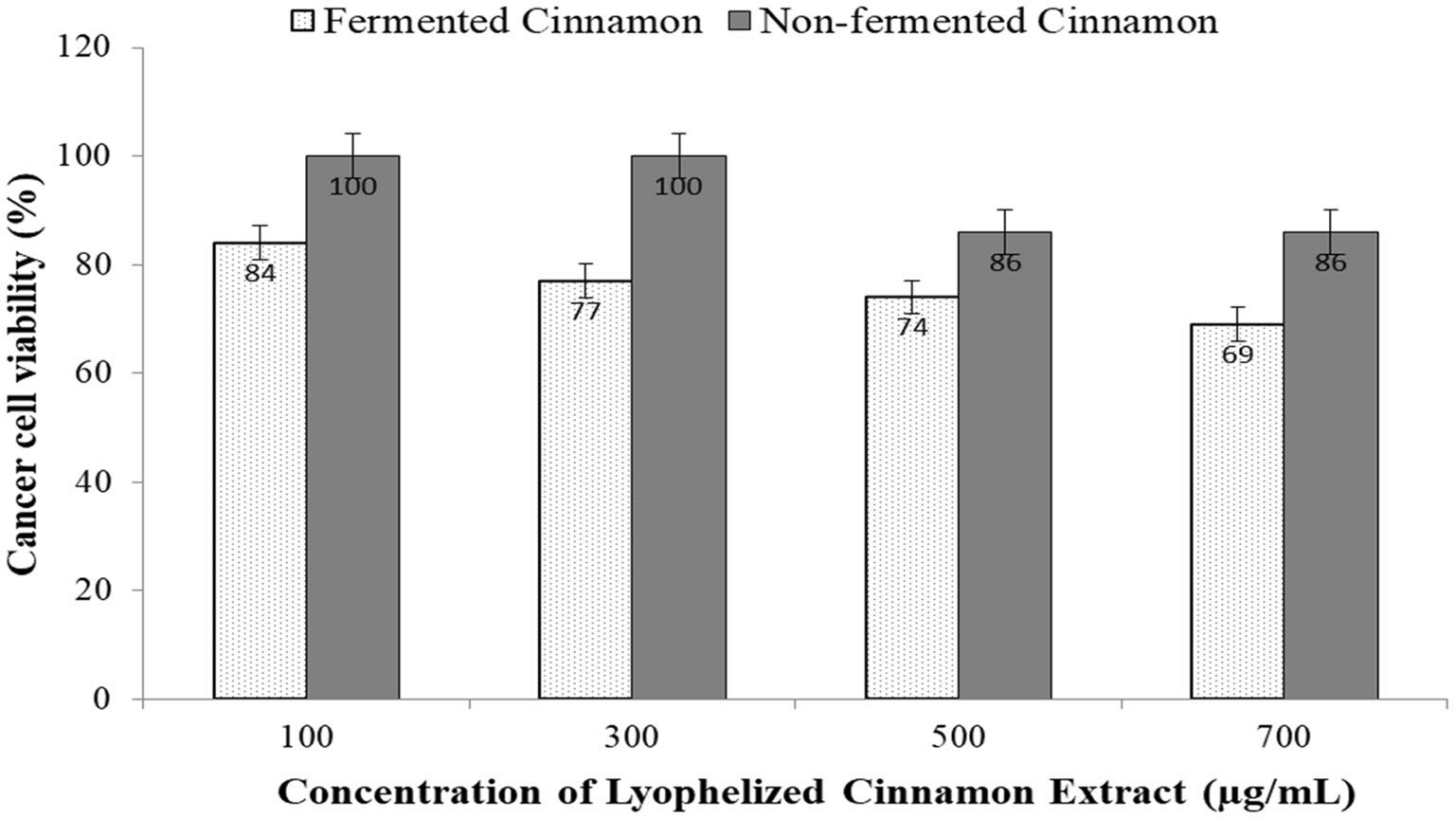
Cancer cell viability after treating with lyophilized extracts of *S. cerevisiae*-fermented and non-fermented cinnamon

Cinnamon has been shown to have anti-cancer properties (Hamidpour et al. 2015), and it could inhibit cancer cell proliferation and increases apoptosis in human oral cancer (Wu et al. 2018). On the other hand, liver cancer is a serious health problem, the fourth-largest cause of cancer-related deaths globally, and the sixth most prevalent cancer (Leone et al. 2021). The most frequent type of liver cancer, hepatocellular carcinoma (HCC), is ranked as the fourth-leading cause of cancer-related death worldwide (Huang et al. 2020). Therefore, it is crucial to understand if fermentation with the yeast *S. cerevisiae* could increase or decrease this activity based on liver cancer cells (Huh7). The obtained results showed that at all concentrations tested, extracts of fermented cinnamon clearly decreased the metastasis of cancer cells compared to the extracts of non-fermented cinnamon (Fig. 4). The fermented cinnamon decreased the cancer cell viability by 31% at the concentration of 700 µg/mL. However, the obtained results was limited to the concentration of 700 µg/mL, and higher concentrations of lyophilized fermented cinnamon extracts may increase the death of cancer cells. Consequently, the fermentation of cinnamon powder with the yeast *S. cerevisiae* successfully increases the anti-cancer activity of cinnamon (Additional file 1: Fig. S3).

### Characterization and identification the products of fermentation processes

Some biologically active chemicals may be created or removed from the fermentation media as a result of fermentation. Therefore, to examine the primary components of cinnamon before and after fermentation of cinnamon with *S. cerevisiae*, thin-layer chromatography was employed. Analysis at Additional file 1: Fig. S4 revealed that one of the components from the fermented cinnamon had vanished. Cinnamaldehyde, an aromatic aldehyde chemical found in cinnamon, may be this substance (Utchariyakiat et al. 2016). Cinnamaldehyde separated on silica gel more quickly than cinnamic acids. Additionally, the oxidation of aldehydes produced carboxylic acids like cinnamic acid. As a result, cinnamaldehyde may oxidize to cinnamic acid during fermentation (Poole and Poole 1994; Dvorackova et al. 2015). Caffeic acid and p-coumaric acid were discovered in both fermented and unfermented cinnamon using thin-layer chromatography. Both of those substances have been identified as natural antioxidants (Kiliç and Yeşiloğlu 2013; Espíndola et al. 2019). TLC analysis is regarded as the first stage of characterization and must be followed by a more advanced analysis, such as HPLC. Therefore, utilizing a number of common antioxidant chemicals, the fermented samples were analyzed using HPLC.

**Fig. 5:**
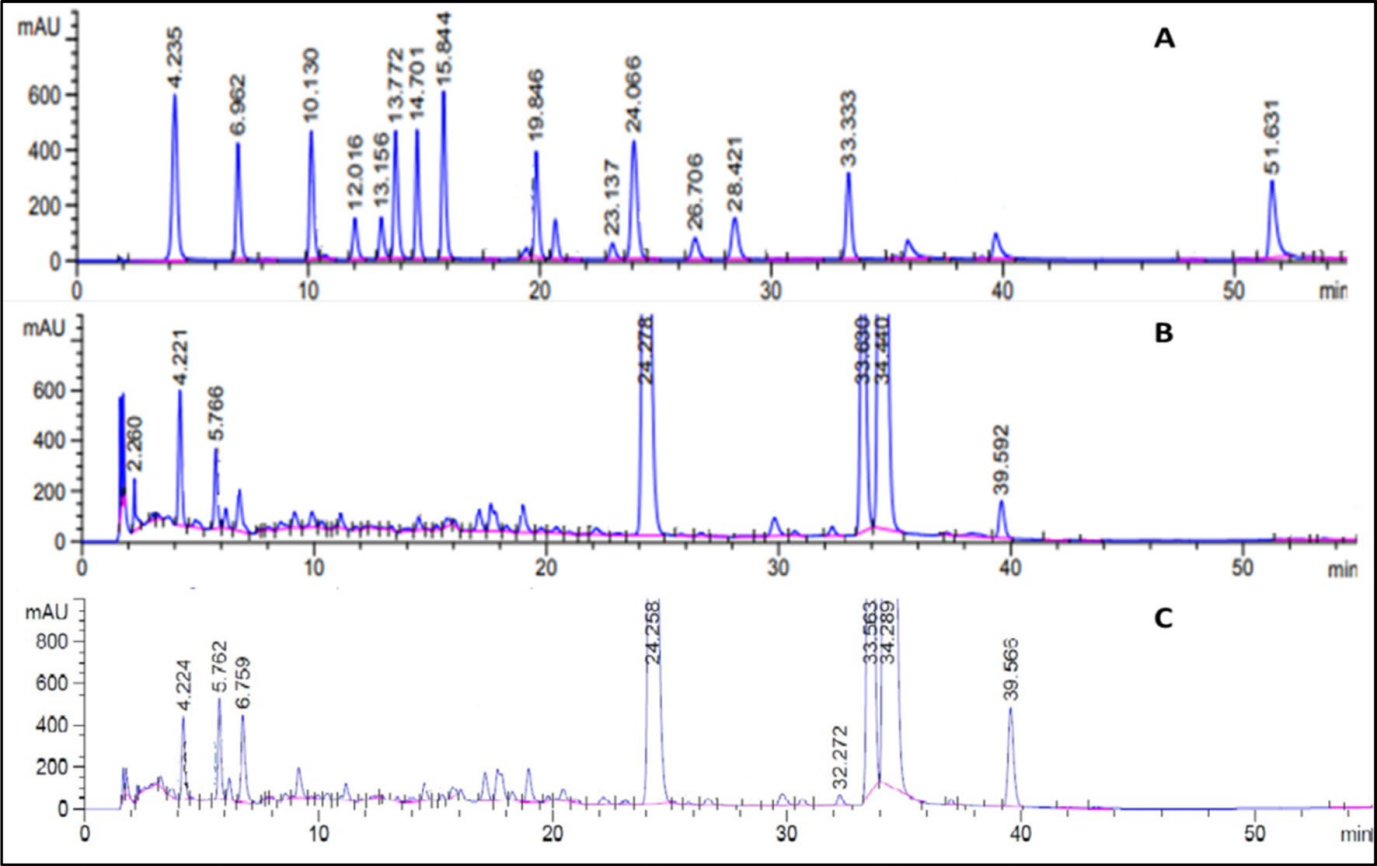
The chromatogram of standards (**A**), non-fermented (**B**), and fermented cinnamon extract (**C**)

The extracts of both fermented and non-fermented cinnamon were subjected to HPLC analysis to identify and measure certain bioactive components (Table 6). Gallic acid, *p*-hydroxybenzoic acid, catechin chlorogenic acid, and protocatechuic acid were all put into HPLC as standards (Fig. 5). Some bioactive compounds have been increased in the lyophilized extract of *S. cerevisiae*-fermented cinnamon. According to HPLC analysis (Table 6), *p*-hydroxybenzoic acid, gentisic acid, catechin, chlorogenic acid, caffeic acid, and syringic acid increases by 116, 33.2, 59.6, 50.6, 1.6, and 16.9%, respectively. The content of antioxidant *p*-hydroxybenzoic acid and gentisic acid may be enhanced because of hydrolysis of benzoic acid during fermentation (Dvorackova et al. 2015; Adhikari et al. 2021). *p*-Hydroxybenzoic acid has antioxidant, and anti-inflammatory characteristics by increasing the expression of antioxidants leading to better plasma lipid profiles (Juurlink et al. 2014). Also, gentisic acid has anti-inflammatory, antigenotoxic, and antimicrobial activities (Abedi et al. 2020). In addition, epicatechin may be converted to the antioxidant catechin during fermentation of cinnamon (Rao and Gan 2014; Fathima and Rao 2016). Additionally, caffeic acid may connect to quinic acid to form chlorogenic acid which has antioxidant activity (Bhattaram et al. 2002; Xu et al. 2012). Moreover, hydrolysis of hydroxycinnamic acid by fermentation may form caffeic acid which is antioxidant and neuroprotective (Sakai and Tsukuba 2015). Furthermore, Janel and Noll (2014) mentioned that lignin can be hydrolyzed to the antioxidant compound syringic acid (Srinivasulu et al. 2018). Fortunately, the decreased compounds, such as gallic acid, may be hydrolyzed to the antioxidant compound catechin (Ahmed and Steppy 2013). Also, protocatechuic acid content was lowered; it may be esterified, and the esterification of hydrophilic phenolic antioxidants increased their antioxidant activity (Reis et al. 2010). According to these results, *S. cerevisiae* fermentation increased the free radical-scavenging activity of cinnamon.

**Table 6:** Differentiation between some bioactive compounds in lyophilized extract of* S. cerevisiae-*fermented and non-fermented cinnamon

Compound		RT (min)	Non-fermented (μg/g)	Fermented (μg/g)	Conversion hypothesis	Biological activity	References
Decreased compounds	Gallic acid	4.2	225.48	162.76	Hydrolysis of gallic acid to form catechin	Antioxidant	Adhikari et al. 2021
	Protocatechuic acid	6.9	520.47	405.04	It may be esterified	Protocatechuic esters has antioxidant activity higher than protocatechuic acid	Reis et al. 2010
Increased compounds	*p*-hydroxybenzoic acid	12	14.65	31.64	Hydrolysis of benzoic acid to form *p*-hydroxybenzoic acid	Promotion of the expression of antioxidant enzymes o	Dvorackova et al. 2015
	Gentisic acid	10.1	40.30	53.66	Hydrolysis of benzoic acid to form gentisic acid	Anti-inflammatory, and antigenotoxic	Abedi et al. 2020
	Cateachin	24	29.40	46.92	Hydrolysis of gallic acid to form catechin	Antioxidant	Rao and Gan, 2014; Fathima and Rao 2016
	Chlorogenic acid	28	8.80	13.25	Quinic acid, and caffeic acid formed chlorogenic acid	Antioxidant	Bhattaram et al. 2002; Xu et al. 2012
	Caffeic acid	40	7.02	7.13	Hydrolysis of hydroxycinnamic acid to form caffeic acid	Antioxidant, and neuroprotective	Sakai and Tsukuba 2015
	Syringic acid	33	55.25	64.59	Degradation of lignin	Antioxidant	Srinivasulu et al. 2018

## Conclusion

Fermenting cinnamon with *S. cerevisiae* can increase its antioxidant and anti-carcinogenic properties without releasing harmful chemicals. The presence of more phenolic components and flavonoids in fermented cinnamon aqueous extracts may be the cause of their improved radical-scavenging capacity. Fermentation process increased *p*-hydroxybenzoic acid, gentisic acid, catechin, and chlorogenic acid which resulted in increasing the antioxidant, anti-inflammatory, antigenotoxic, and neuroprotective activities of cinnamon. Moreover, lyophilized extract of *S. cerevisiae*-fermented cinnamon causes decreasing of human cancer cell viability by 31% at the concentration of 700 µg/mL. The results obtained highlight the significance of the fermenting approach in enhancing the antioxidant and anti-cancer properties of numerous medical plants and herbs.

### Supplementary Information

**Additional file 1: Figure S1.** The standard curve of Gallic acid for determination of total phenolic content. **Figure S2.** The standard curve of quercetin for determination of total flavonoids content. **Figure S3.** Microscopic photo for cancer cells without treatment, treated with fermented cinnamon extract, treated with non-fermented cinnamon extract. **Figure S4.** TLC chromatogram of lyophilized fermented (1) and non-fermented cinnamon (2) extracts compared with caffeic acid (3) and *P*-coumaric acid (4).

## Data Availability

Data will be made available on request.

## Abbreviations

*C**.** cassia*: Cinnamon (*Cinnamomum Cassia*)
*S. cerevisiae*: *Saccharomyces cerevisiae*
DPPH: 2, 2-Diphenyl-1-picrylhydrazyl
ABTS: 2,2′-Azino-bis(3-ethylbenzothiazoline-6-sulfonate diammonium)
H_2_O_2_: Hydrogen peroxide
GAE: Gallic acid equivalent
QE: Quercetin equivalent
TLC: Thin-layer chromatography
HPLC: High-performance liquid chromatography
HCC: Hepatocellular carcinoma
Huh7: Human hepatoma
DMEM: Dulbecco’s modified eagle medium
