# Supplementary material for: Application of environmental-safe fermentation with Saccharomyces cerevisiae for increasing the cinnamon biological activities
Source: Bioresour Bioprocess. 2023 Feb 5;10(1):12. doi: 10.1186/s40643-023-00632-9 (PMC10992612; doi:10.1186/s40643-023-00632-9)
Supplement: Supplementary file 1 — Additional file 1: Figure S1. The standard curve of Gallic acid for determination of total phenolic content. Figure S2. The standard curve of quercetin for determination of total flavonoids content. Figure S3. Microscopic photo for cancer cells without treatment, treated with fermented cinnamon extract, treated with non-fermented cinnamon extract. Figure S4. TLC chromatogram of lyophilized fermented (1) and non-fermented cinnamon (2) extracts compared with caffeic acid (3) and P-coumaric acid (4). [file 40643_2023_632_MOESM1_ESM.docx]

Supplementary Data

**Fig. S1.** The standard curve of Gallic acid for determination of total phenolic content

**Fig. S2.** The standard curve of quercetin for determination of total flavonoids content


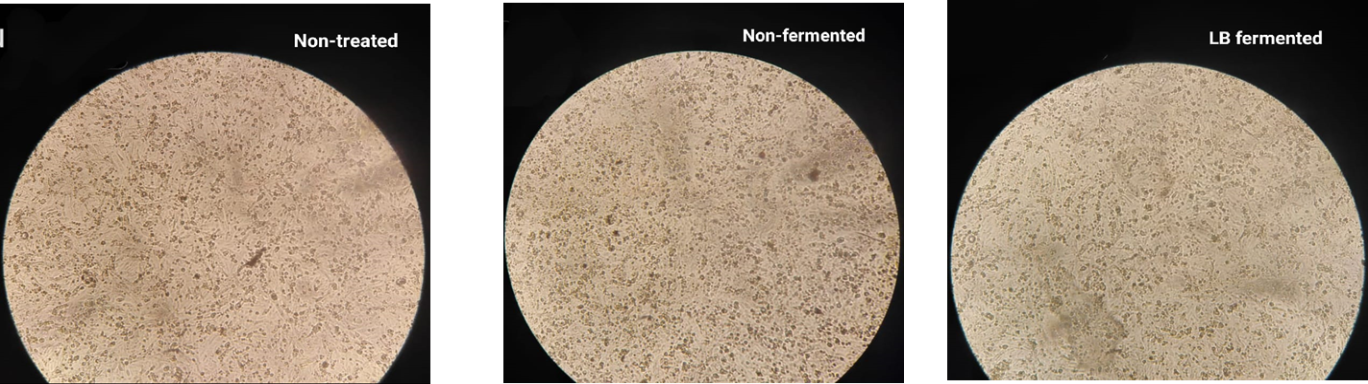
ss

**Fig. S3.** Microscopic photo for cancer cells without treatment, treated with fermented cinnamon extract, treated with non-fermented cinnamon extract


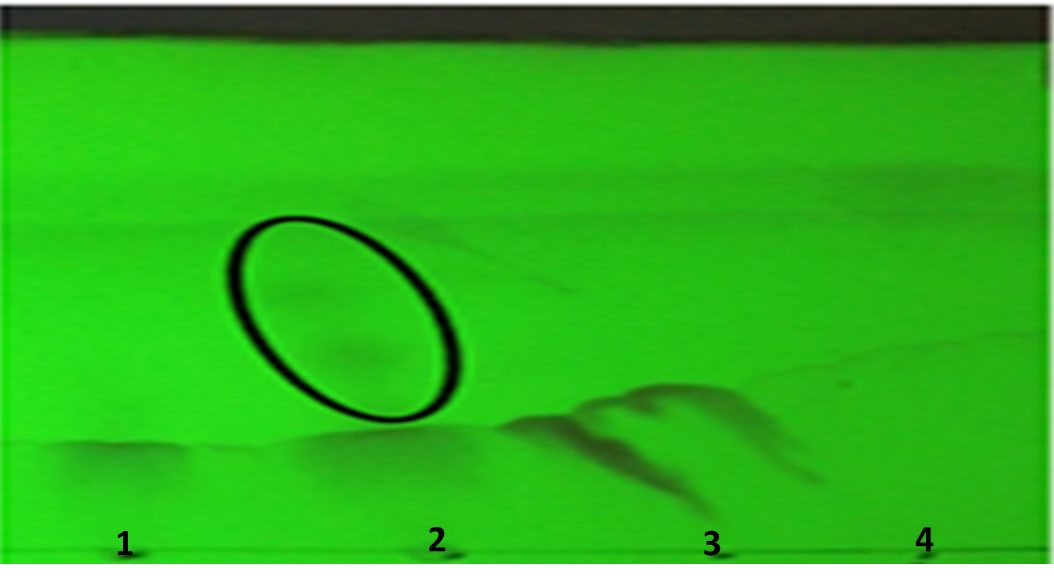
ss

**Fig. S4.** TLC chromatogram of lyophilized fermented (1) and non-fermented cinnamon (2) extracts compared with caffeic acid (3) and *P*-coumaric acid (4).
